# Pickering Emulsions Stabilized by Chitosan/Natural Acacia Gum Biopolymers: Effects of pH and Salt Concentrations

**DOI:** 10.3390/polym14235270

**Published:** 2022-12-02

**Authors:** Ahmad A. Adewunmi, Ahmad Mahboob, Muhammad Shahzad Kamal, Abdullah Sultan

**Affiliations:** 1 Center for Integrative Petroleum Research, College of Petroleum Engineering and Geosciences, King Fahd University of Petroleum and Minerals, Dhahran 31261, Saudi Arabia; 2 Department of Petroleum Engineering, College of Petroleum Engineering and Geosciences, King Fahd University of Petroleum and Minerals, Dhahran 31261, Saudi Arabia

**Keywords:** Pickering emulsion, biopolymer, diesel, chitosan, interfacial tension

## Abstract

In this study, chitosan (CT) and naturally occurring acacia gum (AG) blends were employed as emulsifiers to form a series of emulsions developed from diesel and water. Effects of pH level (3, 5, 10, and 12) and various NaCl salt concentrations (0.25–1%) on the stability, viscosity, and interfacial properties of CT-(1%)/AG-(4%) stabilized Pickering emulsions were evaluated. Bottle test experiment results showed that the stability indexes of the CT/AG emulsions were similar under acidic (3 and 5) and alkaline (10 and 12) pH media. On the other hand, the effects of various NaCl concentrations on the stability of CT-(1%)/AG-(4%) emulsion demonstrated analogous behavior throughout. From all the NaCl concentrations and pH levels examined, viscosities of this emulsion decreased drastically with the increasing shear rate, indicating pseudoplastic fluid with shear thinning characteristics of these emulsions. The viscosity of CT-(1%)/AG-(4%) emulsion increased at a low shear rate and decreased with an increasing shear rate. The presence of NaCl salt and pH change in CT/AG solutions induced a transformation in the interfacial tension (IFT) at the diesel/water interface. Accordingly, the IFT values of diesel/water in the absence of NaCl/CT/AG (without emulsifier and salt) remained fairly constant for a period of 500 s, and its average IFT value was 26.16 mN/m. In the absence of salt, the addition of an emulsifier (CT-(1%)/AG-(4%)) reduced the IFT to 16.69 mN/m. When the salt was added, the IFT values were further reduced to 12.04 mN/m. At low pH, the IFT was higher (17.1 mN/M) compared to the value of the IFT (10.8 mN/M) at high pH. The results obtained will help understand the preparation and performance of such emulsions under different conditions especially relevant to oil field applications.

## 1. Introduction

Ideally, traditional emulsions are stabilized by surfactants or by amphiphilic molecules, such as polysaccharides and proteins with sizes less than 5 nm [1,2]. It was Ramsden and Pickering who reported in the early 1990s an alternative approach to stabilizing emulsions by utilizing small particles in the range of 5 nm to several microns [1]. Already, there is substantial fundamental research work on the thermodynamics and formation of Pickering emulsions. The energy bond of a particle having intermediate wettability at the oil–water interface could be relatively high, thereby making a particle effectively irreversibly adsorbed [3]. The efficiency of any particulate emulsifier largely depends on particle size, particle wettability, particle concentration, particle shape, and interparticle interactions [3]. These particulate emulsifiers could offer advantages over conventional surfactants such as a reduced rate of creaming and promoting stability against coalescence. Biopolymers such as proteins and polysaccharides have been employed in many areas to form stabilized Pickering emulsions [4]. Special interest in the use of biopolymers for emulsion stabilization has received much attention because of their abundance, renewability, biodegradability, and other distinct characteristics such as easy functionalization and high adsorption capacity [5,6]. Emulsion formation using a biopolymer as a surface agent is governed by either direct adsorption to the surface during droplet formation and stabilization, or by interactions with another biopolymer layer or surfactant already situated at the interface [4]. Specifically, chitosan (CT) is a low-toxicity, hydrophilic, and biodegradable polysaccharide having wide usage, especially in the formation of different types of emulsions [7,8]. The emulsifying tendency of CT is linked to its structural heterogeneity. Its main components are heavily hydrophilic, while its residual parts, which are less deacetylated, tend to be hydrophobic [7]. Owing to this, hydrophilic segments of CT localize inside the water whereas the hydrophobic residues are attached to the oil phase. Undoubtedly, CT and its derivatives have been utilized as potential stabilizers to produce a variety of emulsions [7,9,10,11,12,13,14,15,16,17,18,19,20] that were targeted for numerous applications. Acacia gum (AG), also known as “Gum Arabic”, is another favorite polysaccharide that is still receiving little attention concerning its benefits and applicability [21]. AG is commonly utilized as a stabilizer, thickener, emulsifier, surface-finishing material, and flavoring agent [22]. Just like CT, AG has also been investigated as a promising emulsifier for different types of emulsion [2,5,6,8,21,23]. According to the aforementioned studies, both CT and AG have been used independently as stabilizers to produce different emulsion systems. To our knowledge, few studies have investigated the efficacy of CT/AG blends as emulsifiers. In a study conducted by Sharkawy et al. [9], a mixture of synthesized CT/AG nanoparticles was used as a stabilizer to produce Pickering emulsions that may be used in the cosmetics and food industry. In a similar study by these authors, trans-resveratrol topical delivery and photostability were enhanced by using Pickering emulsions produced from CT/AG nanoparticles [24]. Similarly, the stability and structure of whey protein-coated oil-in-water emulsions were modified and improved by studying the influence of CT/AG at different mix ratios and pH of 3 [25]. The current study is believed to be the first of its kind to explore different blends of synthetic CT and naturally occurring AG to form a series of Pickering emulsions. Herein, the impact of pH change and sodium chloride (NaCl) salt on the stability and viscosities of CT/AG Pickering emulsions produced were assessed. The present work reveals how the CT/AG Pickering emulsions behaved in terms of stability and viscosity under acidic and alkaline pH conditions and at various NaCl concentrations. We also discuss the active role of CT/AG emulsifiers and their interactions at the diesel/water interface. The stabilization mechanism of CT/AG emulsifiers at the diesel/water interface was investigated for a fixed period under different pH conditions and NaCl concentrations to gain comprehensive insight into the adsorption kinetics of CT/AG at the interface.

## 2. Experimental Section

### 2.1. Materials and Methods

The acacia gum used was obtained from the bark of the Acacia tree. Particulate matters attached to the gum collected were neatly removed; thereafter, the gum pieces were milled into powder using a laboratory milling device. The powder form was used directly without any further modification. A low-molecular-weight chitosan (CT) powder and sodium chloride salts were purchased from Sigma-Aldrich. Diesel was obtained from a local petrol filling station and distilled water was made available in the laboratory. A 10% vol. of either HCl or NaOH solution was used to adjust emulsions to either an acidic or alkaline medium.

### 2.2. Preparation of Emulsion

The needed amount of CT/AG powder was added inside a 20 mL beaker containing diesel. The mixture of CT/AG and diesel was agitated at a speed of 6000 rpm using a T50 digital ULTRA-TURRAX mixer. After 7 min of agitation, water was added gradually into the mixture using a syringe, and the mixing lasted for another 23 min. The volumes of water and diesel were fixed at 7 mL and 3 mL, respectively, while the concentrations of CT and AG were maintained throughout at 1% and 4%, respectively. A series of emulsions were formed under different pH levels—3, 5, 7 10, and 12—and with various NaCl concentrations (0.25%, 0.5%, 0.75%, and 1%). Thereafter, the emulsions produced were subjected to various characterizations.

### 2.3. Emulsion Stability Test

The emulsion stability index (ESI) of the produced emulsions was studied in ambient conditions using the bottle test technique. The stability was examined visually by monitoring the development of the serum layer at the bottom of the emulsion with respect to time, and ESI was estimated using Equation (1):

(1) $$\mathrm{ESI}=\left[1-\frac{V_{w}}{V_{e}}\right]\times 100$$

where V_W_ and V_e_ represent the volume of separated water and the total volume of emulsion, respectively.

### 2.4. Characterizations

Functional groups of CT and AG were characterized using the Fourier Transform Infrared (FTIR) instrument manufactured by Bruker. A thermogravimetric analyzer (TGA) manufactured by TA Instruments was used to evaluate the thermal stability of CT and AG powders. TGA tests were conducted under a nitrogen atmosphere, at a heating rate of 10 °C/min from 25 °C to 700 °C. Viscosity measurements were carried out using the discovery DHR-3 rheometer manufactured by TA Instruments. The concentric cylinder geometry was employed to investigate the viscosity of emulsion samples at 25 °C. An Olympus microscope with high imaging resolution was used to analyze the dispersion and morphology of emulsions formed. Interfacial tension equipment manufactured by Biolin Scientific was used to determine the interfacial tension (IFT) at the diesel/water interface in the presence and absence of CT/AG mixtures and under varying pH conditions and different NaCl concentrations. Approximately 17 μL of diesel was carefully released from the capillary tube onto the tip of a J-shaped needle inserted into the CT/AG colloidal solution. Likewise, the IFT of diesel/water without CT/AG (i.e., blank sample) was determined. All IFT measurements were conducted at ambient conditions (25 °C) and repeated twice to affirm reproducibility.

## 3. Results and Discussion

### 3.1. Formation of CT/AG Emulsion

In this study, various blends of CT and AG powders were used as emulsifiers to produce emulsions prepared from diesel and distilled water. Figure S1 (supplementary materials) exhibits the FTIR spectra revealing the functional groups in CT and AG. As can be seen in the FTIR spectrum of CT, the prominent characteristic bands shown at 898 cm^−1^ were attributed to the bending O-H vibrations, 1090 cm^−1^ was associated with the C-O-H stretching bonds of CT, 1646 cm^−1^ was attributed to C-O stretching, 2876 cm^−1^ was due to C-H stretching, and 3357 cm^−1^ was due to N–H and O–H stretching. As for the FTIR spectrum of AG, the characteristic bands at 1049 cm^−1^ were believed to be due to C-O stretching of the primary alcohol, the band at 1634 cm^−1^ was associated with the asymmetric and symmetric stretching of the COO− domain, and that at 3308 cm^−1^ was due to hydrogen bonding. All the characteristics of bands mentioned agreed closely with the studies of Olajire and Bamigbade [26], Eddarai et al. [27], Xu et al. [28], Agnihotri et al. [29], and Liu et al. [30]. Figure S2 (supplementary materials) shows the TGA curves describing the thermal stability of CT and AG conducted at room temperature (25 °C) to 700 °C. The slight weight loss around 100 °C was due to the moisture contents in these biopolymers. Afterward, both CT and AG displayed considerable stability until around 255 °C, where the second stage of weight loss was experienced. This second stage of weight loss was due to the decomposition in both structures, and the final stage weight loss experienced above 338 °C was ascribed to depolymerization, dehydration of the monomers, and complete disentanglement of CT/AG molecules [31]. 

Emulsion formation is achieved through synergy between the CT and AG blends. Figure S3 (supplementary materials) demonstrates the possible emulsification mechanism of CT/AG particles. Stage 1 is the initial dispersion of the CT/AG mixture in the diesel phase before contacting water. Following the diesel/water contact, CT/AG particles aligned and adsorbed at the diesel/water interface to diminish the interfacial tension, and stage 2 exhibited how the CT/AG particles formed boundary layers around the diesel droplets (dispersed medium) within the water as the continuous phase. Therefore, all emulsions produced were of diesel-in-water type. The dilution test technique was further employed to confirm the dispersed and continuous media. The interactions that might have occurred between the CT and the AG via hydrogen bonding [32] are shown in Figure S4 (supplementary materials). Moreover, an alteration that might have happened in the hydrophobicity and hydrophilicity [33] of CT/AG biopolymers during blending could have favored emulsion formation between diesel and distilled water.

### 3.2. Effects of pH and Salt on the Stability of CT/AG Pickering Emulsion

**Figure 1:**
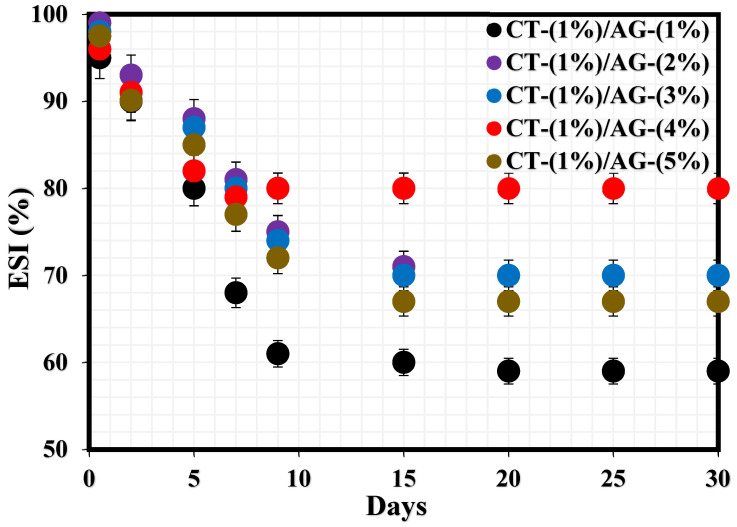
Stability tests of emulsions stabilized by various CT/AG blends.

The stability index of CT/AG emulsions before pH adjustment and in the absence of NaCl salt was monitored for one month using the bottle test technique. Figure 1 shows the percentage emulsion stability index (%ESI) of these emulsions. All the samples experienced a drastic drop in the %ESI within 7–9 days, after which these emulsions remained stable in the remaining days. The %ESI values of 1%-CT/1%-AG, 1%-CT/2%-AG, 1%-CT/3%-AG, 1%-CT/4%-AG, and 1%-CT/5%-AG emulsions after 30 days were determined to be 40.11%, 30.37%, 30.37%, 20.27%, and 33.01%, respectively, indicating that the emulsion filled with 1%-CT/4%-AG emulsifier was the most stable. It also indicates that the stability of these emulsions increased as the concentration of AG increased in the emulsifier blend. The aforementioned stability test revealed that approximately 20% of the water separated from 1%-CT/4%-AG emulsion after 30 days. Therefore, the effects of pH dispersion under acidic and alkaline media and the effects of changing salt concentrations on 1%-CT/4%-AG emulsion were investigated to derive further insight into the stability of this emulsion. 

**Figure 2:**
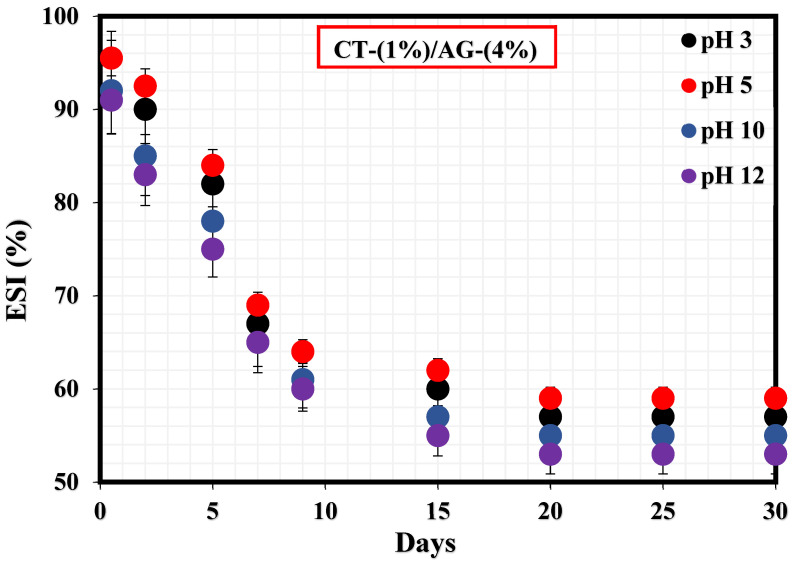
Stability tests of CT/AG Pickering emulsions under different pH levels.

Figure 2 exhibits the %ESI of 1%-CT/4%-AG emulsion under different pH (3, 5, 10, and 12) values. Accordingly, the %ESI values of this emulsion under pH 3, 5, 10, and 12 were estimated to be 43.15%, 41.11%, 45.77%, and 47.19%, respectively. In all cases, the stability indexes of this emulsion were observed to be close to one another under acidic (3 and 5) and alkaline pH (10 and 12) media. Literature work has suggested that closely arranged network structures especially at high pH could impact the network structure and compactness of emulsion [1]. Moreover, these stability results suggest that the adsorption and dispersion [34] of CT/AG emulsifier particles at the diesel/water interface are nearly the same across the pH ranges under investigation.

**Figure 3:**
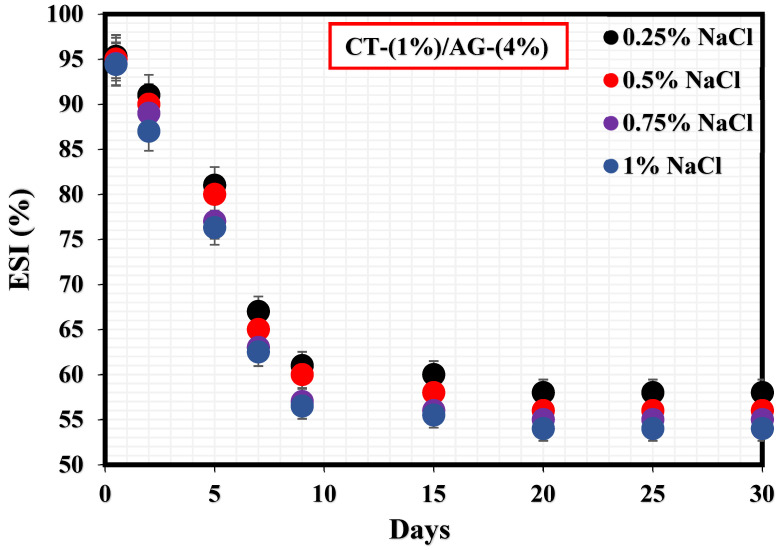
Stability tests of CT/AG Pickering emulsions at varying NaCl concentrations.

Figure 3 shows how NaCl concentrations (0.25–1%) impacted the stability of 1%-CT/4%-AG emulsion. A sharp drop in this emulsion’s stability was observed within 9 days at various NaCl concentrations. The %ESI values of 1%-CT/4%-AG emulsion in the presence of 0.25, 0.5, 0.75, and 1% NaCl were estimated to be 40.1%, 42.8%, 43.3%, and 43.5%, respectively, indicating that 1%-CT/4%-AG emulsion containing 0.25% NaCl exhibited the optimal stability because approximately 40% of water separated from the emulsion after 30 days. The effects of the evaluated NaCl concentrations on the stability of 1%-CT/4%-AG emulsion demonstrated nearly similar characteristics, as the %ESI values after 30 days of monitoring at various NaCl concentrations were close (as stated above), with a slight numerical difference. 

**Figure 4:**
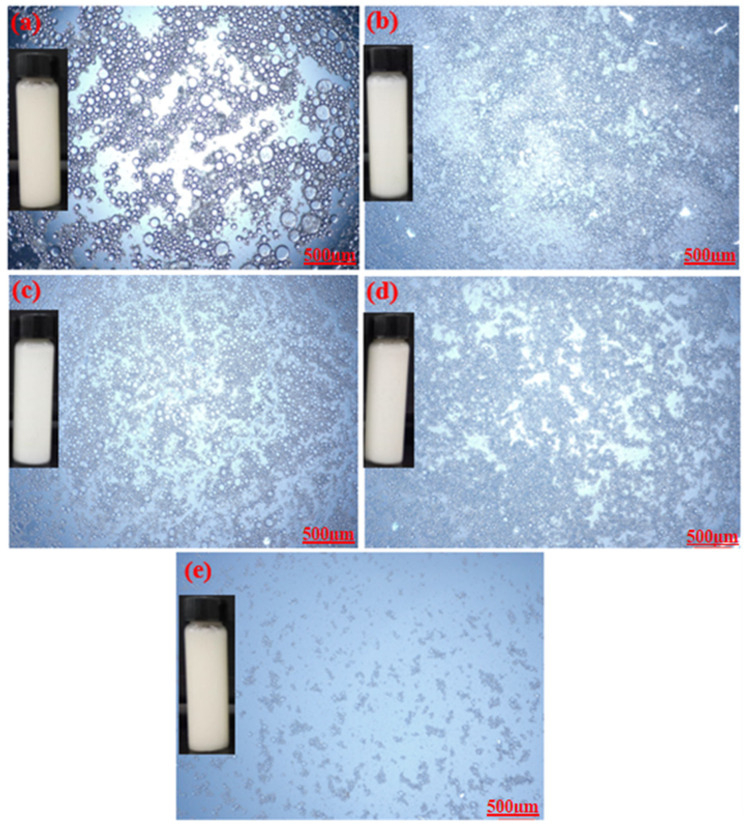
Optical microscopy images of emulsions stabilized by: (**a**) 1%-CT/1%-AG, (**b**) 1%-CT/2%-AG, (**c**) 1%-CT/3%-AG, (**d**) 1%-CT/4%-AG, and (**e**) 1%-CT/5%-AG.

**Figure 5:**
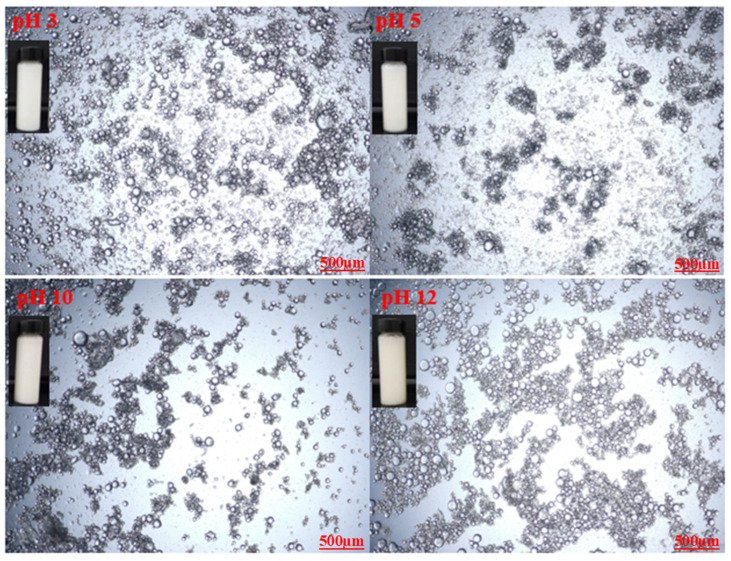
Optical microscopy images of 1%-CT/1%-AG emulsion under different pH levels.

**Figure 6:**
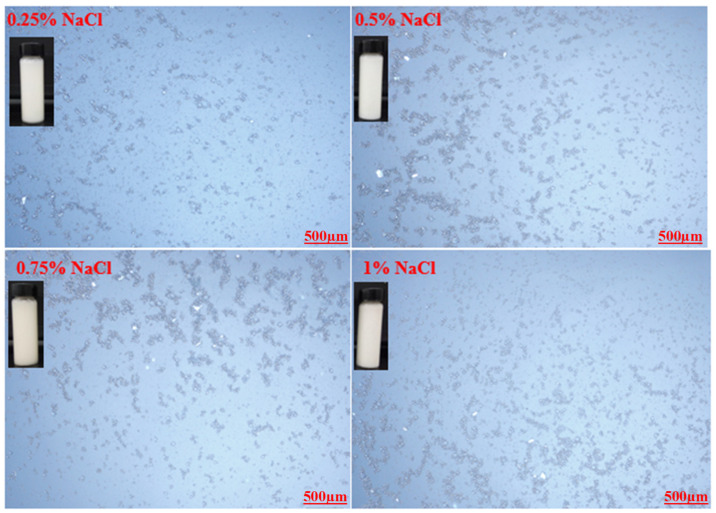
Optical microscopy images of 1%-CT/1%-AG emulsion at various NaCl concentrations.

Optical microscopic images of the prepared emulsions stabilized by various CT/AG blends in the absence of NaCl salt and without pH adjustment are presented in Figure 4. It was observed that the emulsion droplets became smaller with the increasing concentration of AG. Emulsion stabilized by 1%-CT/1%-AG (Figure 4a) had droplet sizes in the range of 0.1–7.5 microns, whereas the droplet sizes of emulsion samples stabilized by 1%-CT/2–5%-AG (Figure 4b–e) were within 0.09–5.7 microns. The optical microscopic images of 1%-CT/4%-AG emulsion under different pH levels and various NaCl concentrations are shown in Figure 5 and Figure 6. The dispersion of emulsion droplets was glaring across the investigated pH ranges. The average droplet sizes of the emulsion at pH 3, 5, 10, and 12 were in the range of 0.18–6.9 microns. Conversely, the optical microscopic images of 1%-CT/4%-AG emulsion containing different NaCl concentrations (0.25, 0.5, 0.75, and 1%) displayed similarities (Figure 6) to the same emulsion (Figure 4d) where NaCl was not incorporated, with average emulsion droplet sizes of around 0.1–5.9 microns across the various NaCl concentrations. Following these observations, the influence of pH and NaCl salt on the CT/AG Pickering emulsion was further quantified via rheology and interfacial analysis. 

### 3.3. Effect of Salt and pH on the Viscosity of CT/AG Pickering Emulsion

**Figure 7:**
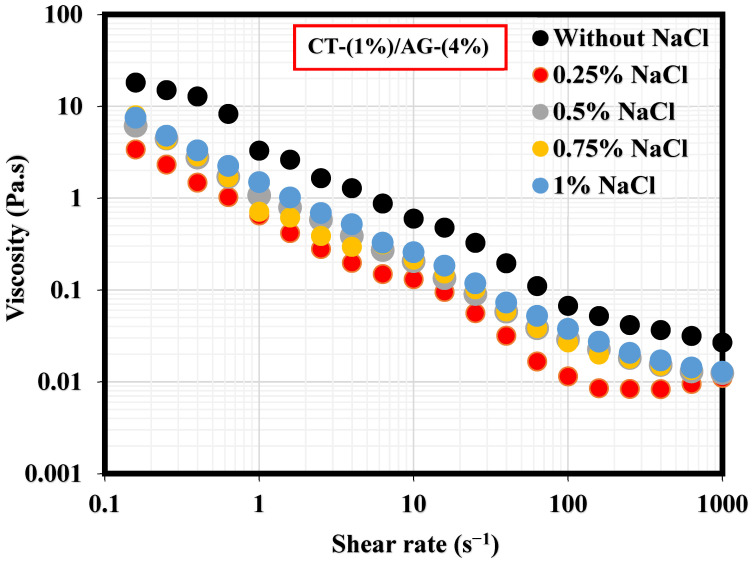
Viscosity behavior of 1%-CT/4%-AG emulsion at various NaCl concentrations.

**Figure 8:**
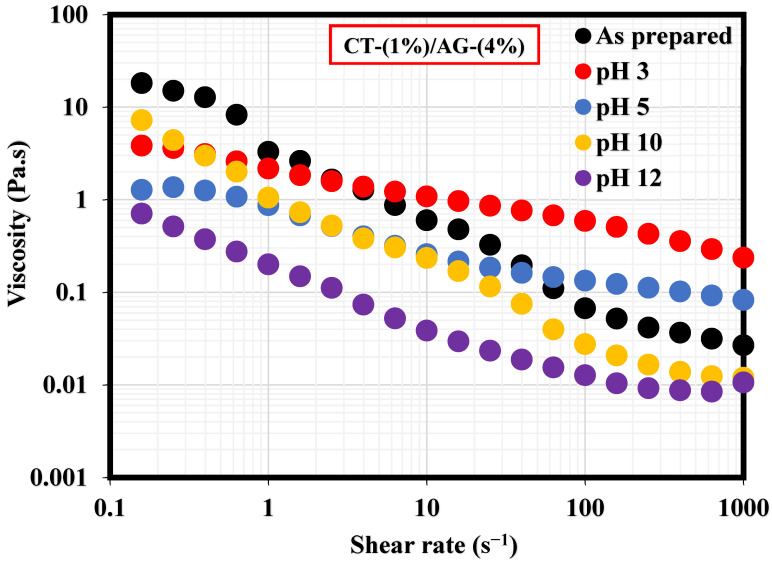
Viscosity behavior of 1%-CT/4%-AG emulsion under different pH levels.

**Figure 9:**
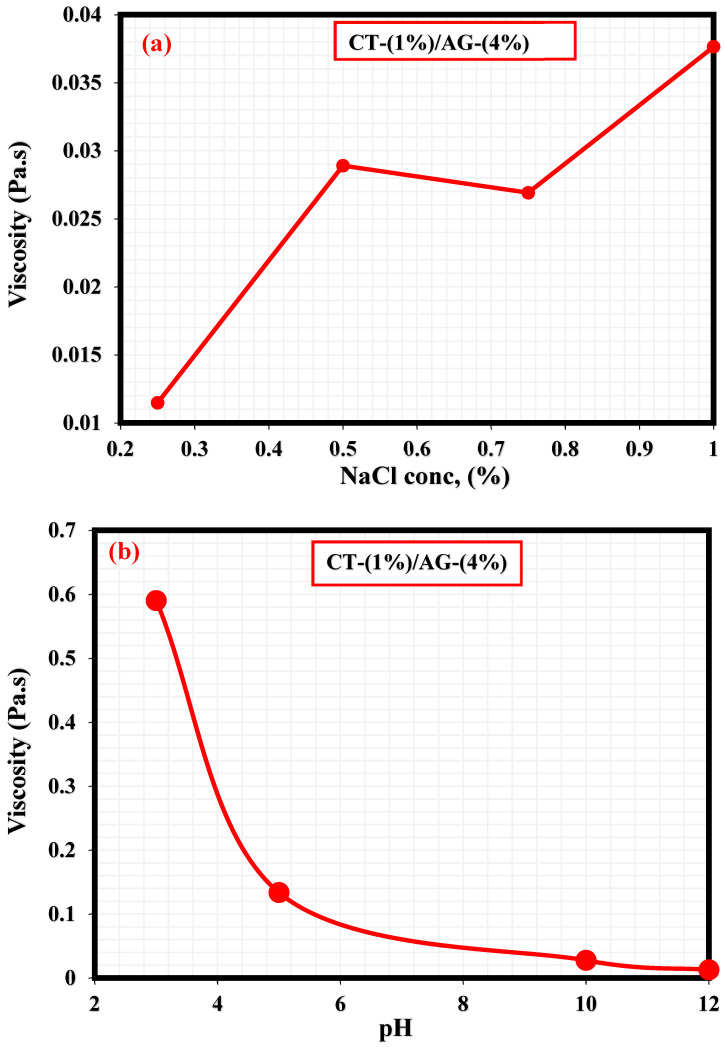
(**a**) Viscosity dependence of 1%-CT/4%-AG emulsion at various NaCl concentrations. (**b**) Viscosity dependence of 1%-CT/4%-AG emulsion under pH at constant shear rate.

A literature study revealed that the viscosity of an emulsion has a pronounced effect on the migration of the emulsifier to the interface of two immiscible fluids [35]. This section, therefore, elucidates the effect of NaCl concentrations and pH on 1%-CT/4%-AG emulsion. From all the NaCl concentrations (0.25–1%) and pH levels (3, 5, 10, and 12) examined, viscosities of 1%-CT/4%-AG emulsion decreased drastically with the increasing shear rate (Figure 7 and Figure 8), indicating that 1%-CT/4%-AG emulsion behaved as a pseudoplastic fluid with shear thinning characteristics [35]. This phenomenon could be linked to the fact that higher shear strain would lead to further elongation, network destabilization, and deformation of the emulsion structure. However, the viscosity reduction of 1%-CT/4%-AG emulsion became more pronounced at 1000 s^−1^ (~0.01 Pa.s for every NaCl tested). On the other hand, the influence of pH change from acidic to alkaline medium was noticed on the viscosity of 1%-CT/4%-AG emulsion, especially at higher shear rates. Under pH 3, 5, 10, and 12, the viscosities of 1%-CT/4%-AG emulsion at 1000 s^−1^ were 0.24 Pa.s, 0.08 Pa.s, 0.01 Pa.s, and 0.01 Pa.s, respectively. The inference from this observation is that the 1%-CT/4%-AG emulsion’s network could behave differently under an acidic medium, and that it is capable of exhibiting a similar network structure under an alkaline medium when the shear rate is presumably high (≥100 s^−1^). The viscosity dependence of this emulsion at a fixed shear rate (100 s^−1^) for 0.25–1% NaCl concentrations and pH 3, 5, 10, and 12 is displayed in Figure 9a,b. The data shown in Figure 9a demonstrate that under a constant shear rate, the emulsion’s viscosity increases for 0.25–0.5% NaCl and drops slightly for 0.75% NaCl, and then increases with 1% NaCl. The change or slight drop in the emulsion’s viscosity at 0.75% NaCl could be attributed to changes in emulsion morphology and structure. Conversely, the viscosity of 1%-CT/4%-AG emulsion under a fixed shear rate decreased progressively at pH 3, 5, 10, and 12, suggesting that this emulsion displayed a structural change under acidic and alkaline mediums. Briefly, the viscosity was observed to have increased at a low shear rate and decreased as the shear rate increased in these emulsions at various NaCl concentrations and pH levels. 

### 3.4. Effect of Salt and pH on the Adsorption of CT/AG at Diesel/Water Interface

**Figure 10:**
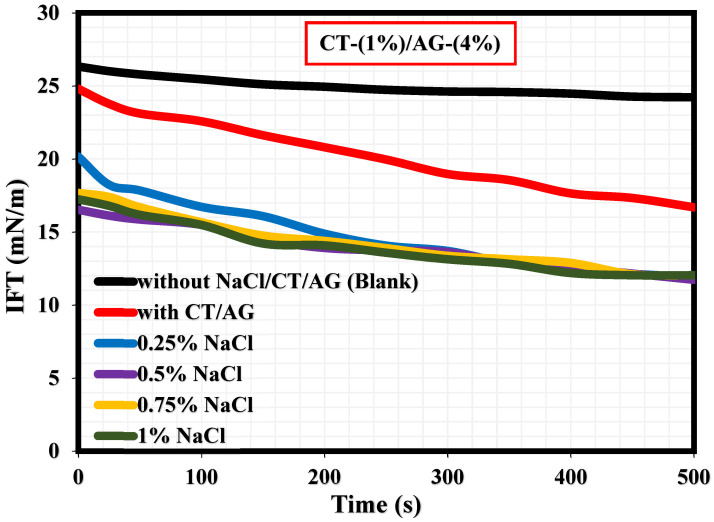
Effects of NaCl salt on the dynamic IFT at diesel/water interface.

**Figure 11:**
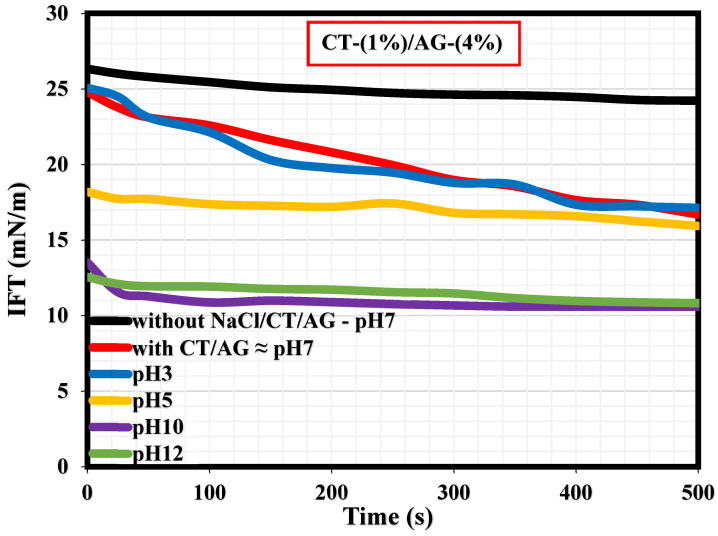
Effects of pH on the dynamic IFT at the diesel/water interface.

A literature survey revealed that only a few studies have actually reported the interfacial tension (IFT) measurements explaining the adsorption kinetics at the diesel/water interface in the presence and absence of surfactants [36,37,38,39,40]. To our knowledge, the current study would probably be the first research endeavor reporting the IFT measurements of migration of CT/AG emulsifiers at the diesel/water interface and how NaCl salt and pH affect the CT/AG particle adsorption at the diesel/water interface. Figure 10 shows the dynamic IFT measurements revealing the adsorption kinetics at the diesel/water interface with and without 1%-CT/4%-AG, and also in the presence of various NaCl concentrations dissolved in 1%-CT/4%-AG solution. Accordingly, the IFT values of diesel/water in the absence of NaCl/CT/AG remained fairly constant for a period of 500 s, and the average IFT value was 26.16 mN/m. This IFT value of diesel/water in the absence of NaCl/CT/AG is in agreement with the value reported in a previous study [37]. Following the introduction of CT/AG and 0.25–1% NaCl concentrations in water, drastic reductions in IFT at the diesel/water interface were evident, and the reduction was more pronounced in the presence of salt. Certainly, the dispersion of CT/AG in water and various NaCl solutions triggered interfacial tension reduction at the diesel/water interface. The IFT at the diesel/water interface decreased to approximately 16.69 mN/m in the presence of CT/AG alone and to 12.04 mN/m following the addition of different NaCl concentrations after 500 s. Shown in Figure 11 are the dynamic IFT measurements, elucidating the effect of different pH levels of dispersed 1%-CT/4%-AG solution at the diesel/water interface. Evidently, dispersed 1%-CT/4%-AG solution at pH 3, 5, 10, and 12 was examined, causing further IFT reduction at the diesel/water interface compared to the IFT response of 1%-CT/4%-AG solution under pH 7. Dispersion of 1%-CT/4%-AG solution under pH 3 and 5 led to IFT reduction to 17.13 mN/m and 15.93 mN/m, respectively, while the dispersion of 1%-CT/4%-AG in water under pH 10 and 12 induced almost the same IFT value of approximately 10.82 mN/m after 500 s.

## 4. Conclusions

Herein, the effects of NaCl salt concentrations (0.25–1%) and pH levels (3, 5, 10, and 12) were assessed on the stability, viscosity, and interfacial properties of Pickering emulsions produced from a blend of chitosan (CT) and natural acacia gum (AG) as emulsifiers. Preliminary tests without changing pH and in the absence of salt revealed that 1%-CT/4%-AG emulsion had optimal stability among all the emulsions prepared. Hence, the impact of NaCl salt and pH was focused on 1%-CT/4%-AG emulsion. Accordingly, stability index tests showed that the 1%-CT/4%-AG emulsion stability is nearly similar under acidic and alkaline pH. Conversely, the effects of various NaCl concentrations on the stability of 1%-CT/4%-AG emulsion revealed almost similar behavior throughout. Under the influence of various NaCl salt concentrations and different pH levels, viscosities of 1%-CT/4%-AG emulsion diminished with the increasing shear rate. At a low shear rate, the emulsion’s viscosity increased, and it decreased at a high shear rate domain. The interfacial tension (IFT) measurements of diesel/water in the absence of NaCl/CT/AG were fairly constant for a period of 500 s and the average IFT value was 26.16 mN/m, while the IFT at the diesel/water interface in the presence of 1%-CT/4%-AG and various NaCl salt concentrations decreased to approximately 16.69 mN/m and 12.04 mN/m, respectively. Dispersion of 1%-CT/4%-AG in water at pH 3 and 5 caused the IFT reduction at the diesel/water interface to 17.13 mN/m and 15.93 mN/m, respectively, whereas 1%-CT/4%-AG dispersion in water under pH 10 and 12 produced nearly the same IFT value of approximately 10.82 mN/m.

## Data Availability

The data presented in this study are available on request from the corresponding author.

## Supplementary Materials

The following supporting information can be downloaded at: https://www.mdpi.com/article/10.3390/polym14235270/s1, Figure S1: FTIR spectra of chitosan (CT) and acacia gum (AG); Figure S2: TGA curves of CT and AG; Figure S3: Schematic diagram illustrating the emulsification mechanism of CT/AG blends; Figure S4: The interactions between the CT/AG blend.
