# Supplementary material for: Pickering Emulsions Stabilized by Chitosan/Natural Acacia Gum Biopolymers: Effects of pH and Salt Concentrations
Source: Polymers (Basel). 2022 Dec 2;14(23):5270. doi: 10.3390/polym14235270 (PMC9738950; doi:10.3390/polym14235270)
Supplement: Supplementary file 1 [file polymers-14-05270-s001.zip › polymers-2046784-supplementary.pdf]

Pickering Emulsions Stabilized by Chitosan/Natural Acacia Gum Biopolymers: Effects of pH and Salt Concentrations

Ahmad A. Adewunmi [1], Ahmad Mahboob [1], Muhammad Shahzad Kamal [1,*] and Abdullah Sultan [2,*]

[1] Center for Integrative Petroleum Research, College of Petroleum Engineering and Geosciences, King Fahd University of Petroleum and Minerals, Dhahran 31261, Saudi Arabia

[2] Department of Petroleum Engineering, College of Petroleum Engineering and Geosciences, King Fahd University of Petroleum and Minerals, Dhahran 31261, Saudi Arabia

*Correspondence: shahzadmalik@kfupm.edu.sa; sultanas@kfupm.edu.sa Tel: +966-013-8608513

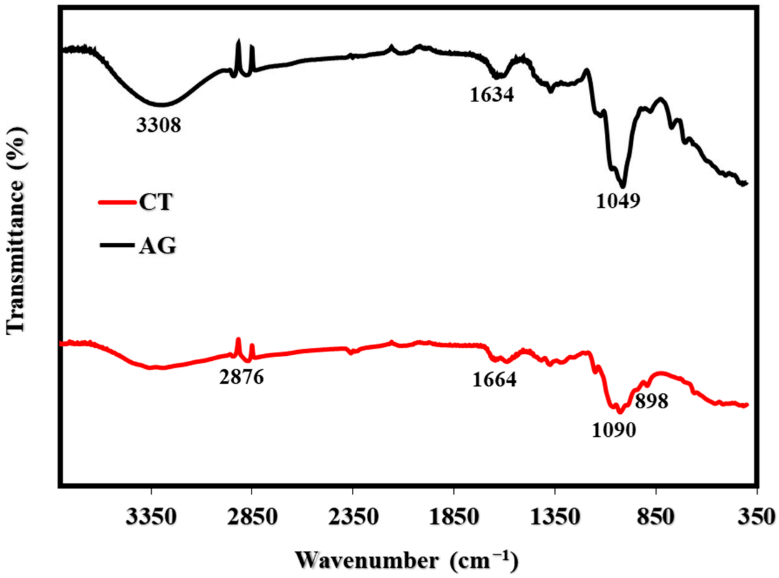

Figure S1. FTIR spectra of chitosan (CT) and acacia gum (AG).

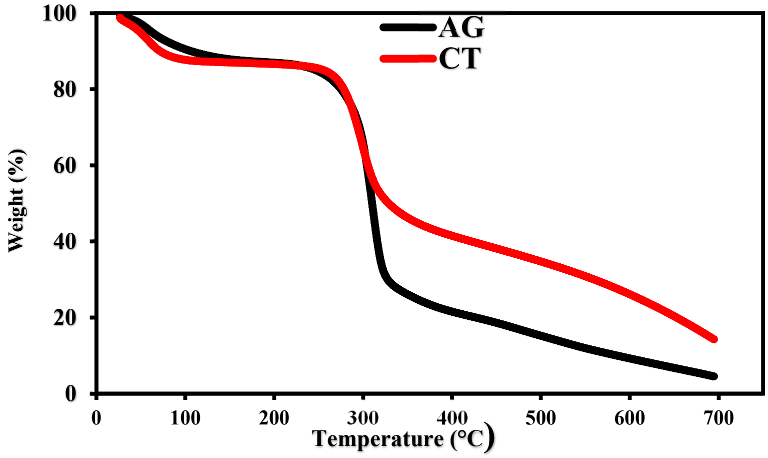

Figure S2. TGA curves of CT and AG.

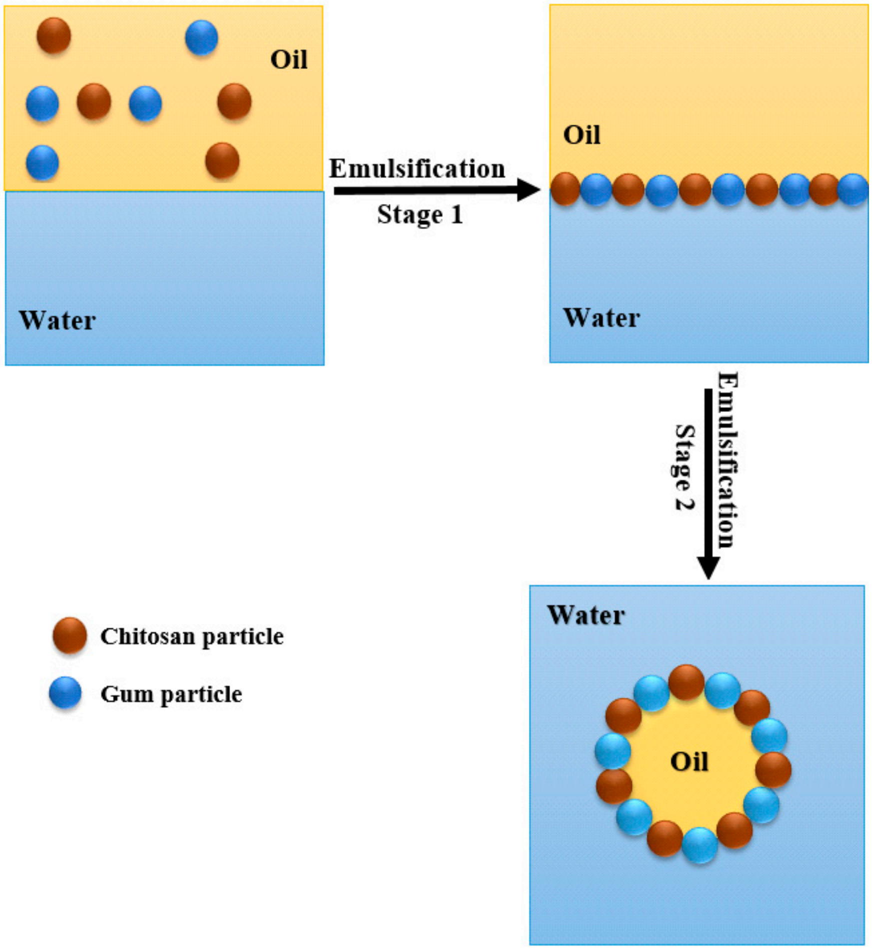

Figure S3. Schematic diagram illustrating the emulsification mechanism of CT/AG blends.

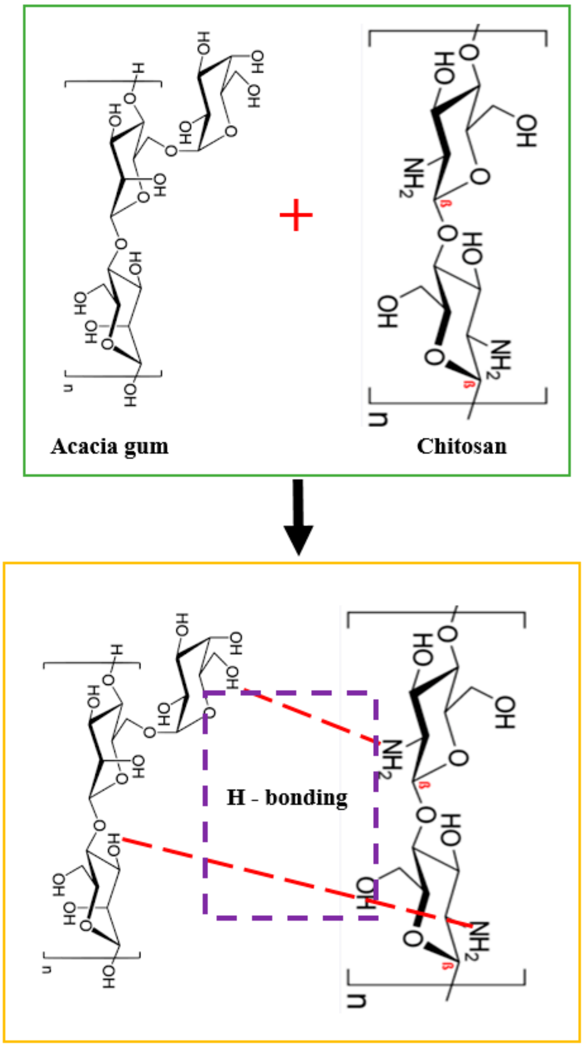

Figure S4. The interactions between the CT/AG blend.
